# Unraveling the Mechanisms of Madecassoside Derivatives in Wound Healing: Network Pharmacology and Experimental Validation

**DOI:** 10.3390/ph18091292

**Published:** 2025-08-28

**Authors:** Jing Liu, Yuanyuan Li, Cheng Yang, Bingtian Zhao

**Affiliations:** Key Laboratory of Synthetic and Biological Colloids, Ministry of Education, School of Chemical and Material Engineering, Jiangnan University, Wuxi 214122, China; lj15735649029@163.com (J.L.); liyuanyuan0918@163.com (Y.L.)

**Keywords:** madecassoside, biomodification, wound healing, oxidative stress, network pharmacology

## Abstract

**Background:** Madecassoside is widely utilized in wound healing due to its multiple physiological activities. However, its limited bioavailability and solubility hinder its clinical application. Enzymatic hydrolysis has been employed to enhance the bioavailability and bioactivity of natural products, but its potential for modifying madecassoside remains unexplored. **Methods:** In this study, we prepared MA1G and MA2G through enzymatic hydrolysis, inspired by the metabolic processes of madecassoside. Network pharmacology was employed to investigate the mechanisms of these madecassoside derivatives (MDs) in wound healing, and molecular docking was performed to evaluate their binding affinities. Transdermal permeation studies, scratch assays, and antioxidant and anti-inflammatory tests were conducted to characterize the biological properties and activities of MDs. **Results:** Network pharmacology identified TLR4, NF-κB, and STAT3 as key targets for wound healing, and the MDs inhibited the expression of these proteins in vitro. Additionally, the results demonstrated that MDs exhibited robust reactive oxygen species (ROS) scavenging activity (43.05–147.50% reduction) and significantly enhanced cell migration (36.76–77.28% increase). Notably, the biomodified MA2G showed superior transdermal permeability and biological activities. **Conclusions:** This paper represents the first report directly comparing the biological activities of the parent compound (madecassoside) and its metabolites while simultaneously proposing a novel therapeutic strategy for wound healing.

## 1. Introduction

Skin wounds represent a significant clinical challenge, with a significant negative impact on patients’ quality of life, as well as a persistent and heavy economic burden [1]. Physiological wound healing typically progresses through sequential phases of hemostasis, inflammatory response, and tissue proliferation [2]. However, persistent microbial colonization and sustained ischemia can disrupt this process by inducing excessive reactive oxygen species (ROS) accumulation and pro-inflammatory cytokine release, thereby impairing cutaneous repair [3,4]. Meanwhile, Toll-like receptor 4 (TLR4), nuclear factor kappa B (NF-κB), and signal transducer and activator of transcription 3 (STAT3) are key regulators in wound healing, with TLR4 activating an inflammatory cascade upon recognizing damage-associated molecular patterns, leading to NF-κB activation and pro-inflammatory cytokine expression [5]. STAT3, essential for cell proliferation and migration, further promotes tissue regeneration [6]. The dysregulation of these pathways impairs wound healing, as observed in chronic wounds with excessive inflammation and delayed repair [7,8]. Consequently, targeting oxidative stress and inflammation represents a promising strategy for wound healing. In recent years, carbon quantum dots, nanozymes, and dopamine have been explored for their potential to scavenge excess ROS in wound healing [9,10,11]. However, these materials have problems with storage stability, production complexity, and long-term safety. Thus, it remains critical to identify safer, more stable, and effective therapeutic alternatives for wound healing.

*Centella asiatica* (CA), a medicinal plant known for its wound-healing properties, has gained considerable attention in recent years for its potential therapeutic effects in various inflammatory diseases [12]. CA contains an array of bioactive compounds, including triterpenes, volatile oils, alkaloids, and flavonoids, which contribute to its diverse pharmacological activities [13]. Among these, triterpenoid saponins such as madecassoside and asiaticoside represent important secondary metabolites. The biosynthesis of these secondary metabolites involves complex sugar chain assembly catalyzed by glycosyltransferases, highlighting their structural complexity [14]. Recent studies have highlighted the multifaceted roles of madecassoside in skin repair, including promoting wound healing, reducing inflammation, and protecting against oxidative stress [15,16]. Given the central role of oxidative stress and inflammation in wound healing, madecassoside has emerged as a promising candidate for wound healing. It is hypothesized that madecassoside may exert its therapeutic effects through the modulation of the oxidative stress and inflammatory network, which may accelerate skin repair.

Although madecassoside has shown significant pharmacological activities, its low solubility and bioavailability greatly limit its development and application [17]. These pharmacokinetic challenges hinder its therapeutic potential and necessitate the development of strategies to improve its absorption and efficacy. Interestingly, a previous study used a rat model to explore the pharmacokinetics of madecassoside and found that madecassoside can be progressively deglycosylated and degraded in vivo to produce three triterpenoids containing different glycosides [18]. Similarly, ginsenoside Rg3 has been reported to undergo deglycosylation in rats to generate the epimers ginsenoside Rh2 (Rh2) and protopanaxadiol (PPD), and its metabolites (PPD and Rh2) have been found to exhibit stronger anticancer activity [19]. These studies revealed that the metabolites of parental compounds may exert better potency due to the reduction of steric hindrance and the improvement of bioavailability [20]. However, the specific mechanisms by which madecassoside’s metabolites enhance bioactivity remain poorly understood, and the exact active components in these metabolites have yet to be identified. This knowledge gap limits the development of more effective therapeutic strategies based on the metabolites of madecassoside. Therefore, it is crucial to systematically investigate the efficacy and mechanisms of these metabolites to identify their active components. It is hypothesized that the exogenous deglycosylated metabolites of madecassoside may exhibit enhanced bioactivity. Nonetheless, directly isolating these metabolites from biological systems remains challenging, further complicating the exploration of their therapeutic potential. Recently, enzyme catalysis in vitro and microbial transformation methods have been widely used for the structural modification of natural products to enhance their bioavailability, stability, and biological activity [21,22]. The enzymatic method is widely used for the glycosylation modification of compounds due to its mildness, high selectivity, high efficiency, and easy separation of products. However, the structural modification of natural triterpenoids from CA by enzymatic hydrolysis has not been studied. Thus, inspired by the drug metabolic processes wherein madecassoside undergoes deglycosylation in vivo, glycosidases have been utilized to prepare its corresponding deglycosylated metabolites.

In this study, we aimed to prepare the deglycosylated metabolites of madecassoside through enzymatic hydrolysis and explore their potential mechanisms in the treatment of wound healing. Network pharmacology was utilized to predict the key signaling pathways involved in the therapeutic effects of madecassoside and its metabolites. Meanwhile, the efficiency variations among MDs were assessed through experiments in vitro and in vivo, confirming their potential mechanisms. This paper highlights the potential of enzyme-catalyzed modifications for enhancing the pharmacological properties of natural products, suggesting that this approach may provide a new molecular design platform for the innovative approaches in the management of wound healing.

## 2. Results

### 2.1. Preparation of MA2G and MA1G

**Figure 1:**
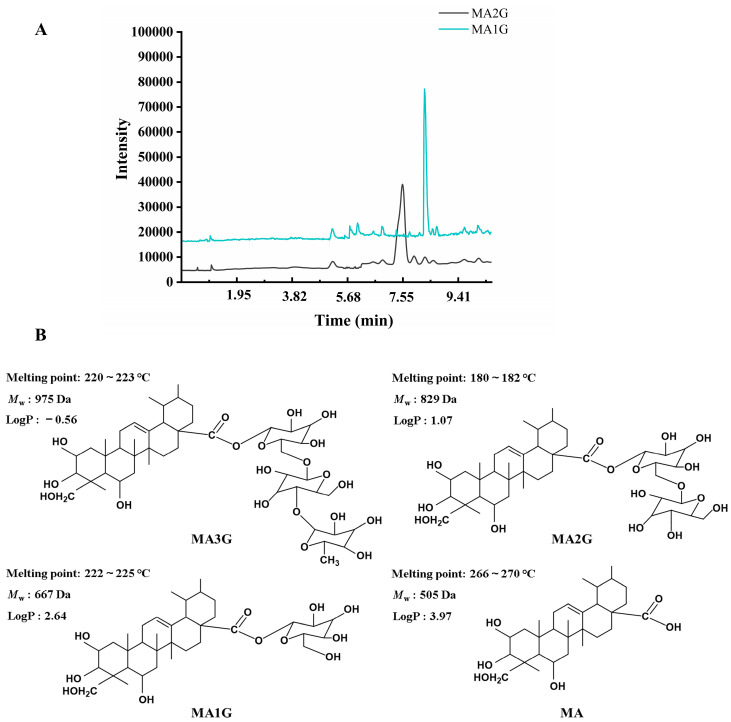
Analysis of MA3G enzymatic hydrolysis products (MA1G and MA2G). (**A**) Total ion chromatograms of MA2G and MA1G. (**B**) The chemical structure, melting point, molecular weight (*M*_w_), and partition coefficient (LogP) of MDs.

The enzymatic hydrolysis products of MA3G were identified using LC-MS (Figure 1A and Figure S1), and the ion fragmentation data are summarized in Table 1. The mass spectrum analysis revealed that the parent ion peaks for MA2G and MA1G were observed at *m*/*z* 829 and *m*/*z* 667, respectively. The difference of 147 Da between MA3G and MA2G corresponded to the loss of one sugar unit while the difference of 162 Da between MA2G and MA1G corresponded to the removal of a second sugar unit. These findings were consistent with the stepwise enzymatic cleavage of MA3G, where one and two sugar units were sequentially removed to generate MA2G and MA1G. These results provide compelling evidence that MA3G was successfully enzymatically degraded into its metabolic products, MA2G and MA1G, confirming the effectiveness of the hydrolysis process. Additionally, the structure of the MDs is illustrated in Figure 1B, which clearly demonstrates the stepwise removal of sugar units from MA3G, leading to its transformation into the metabolites MA2G, MA1G, and MA. Furthermore, HPLC analysis confirmed that the purity of MA1G and MA2G exceeded 95% (Figure S2).

### 2.2. Skin Penetration Test for MDs

**Figure 2:**
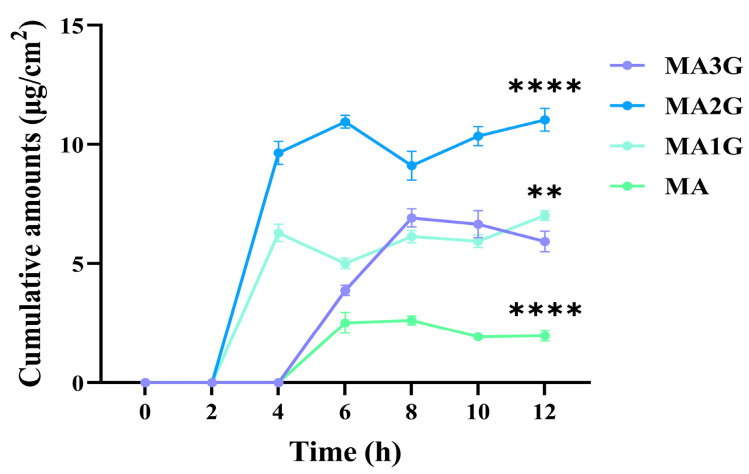
Cumulative permeation amounts of MDs through Strat-M^®^ membranes at different time points (0–12 h) under 37 °C. Data are expressed as mean values ± SD (n = 3). ** *p* < 0.01 and **** *p* < 0.0001 vs. MA3G group.

Penetration testing is essential for the exploration of the physical and chemical properties of drugs, which provides a reference for drug absorption, distribution, and clinical development. Strat-M^®^ membrane is an engineered biomimetic material that mimics the layered structure and lipid chemical composition of human skin [23], which can highly replicate the permeability properties of similar skin in vitro penetration tests. To elucidate the drug properties of MDs, Strat-M^®^ membranes were applied to investigate the transdermal efficiency of MDs to characterize bioavailability. As shown in Figure 2, neither MA3G (0 μg/cm^2^) nor MA (0 μg/cm^2^) penetrated into the skin for 4 h, but MA1G (6.27 μg/cm^2^) and MA2G (9.65 μg/cm^2^) had a significant cumulative effect in the skin during this time. From 4 h to 8 h, the penetration rate of MA3G (6.91 μg/cm^2^) increased significantly, but MA (2.62 μg/cm^2^) still showed a weak skin accumulation effect. After 8 h, the skin penetration rate of all MDs showed their respective plateaus, reaching the highest drug accumulation. The results showed that the degree of glycosylation of drugs significantly affected their permeation kinetics, and the moderately glycosylated MA1G and MA2G showed good skin permeability due to their excellent physicochemical properties.

### 2.3. Network Construction and Enrichment Analysis of Targets

**Figure 3:**
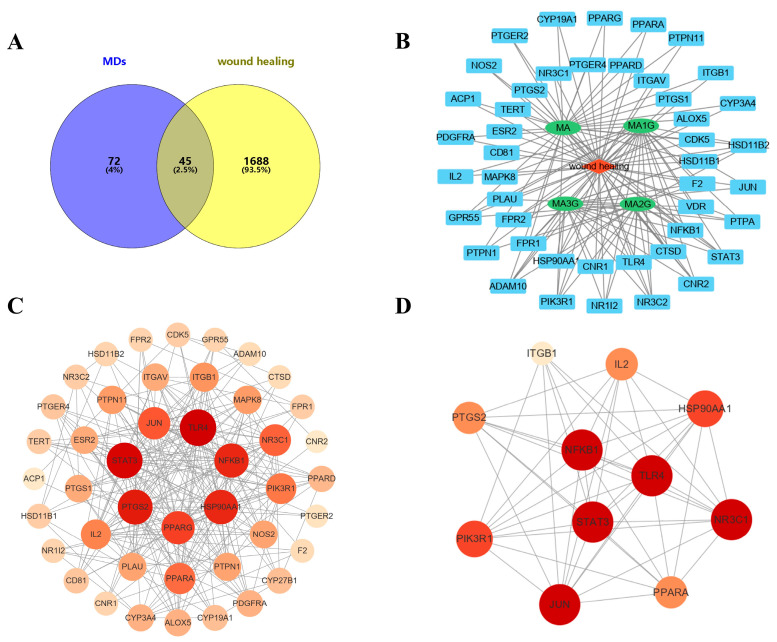
Exploration of the potential mechanism of MDs on wound healing (WH) based on the network pharmacology. (**A**) Venn diagram of 45 common targets between WH and MDs. (**B**) Component–target–disease network. PPI network of common targets (**C**) and core targets (**D**).

To further explore the mechanism of MDs in improving wound healing, network pharmacology was used to analyze the core targets and pathways of MDs. The 117 targets for MDs were identified through TCMSP and SwissTargetPrediction while 1733 disease-related targets for wound healing were obtained (Figure 3A). Finally, 45 intersectional targets between disease and compounds were obtained by a Venn diagram. The MDs–target–wound healing network was obtained by Cytoscape 3.9. (Figure 3B); the results showed that the protein targets of MDs were increased with the gradual decrease in glycans at C28, which may be attributed to the increased molecular chain flexibility of the deglycosylated compound. The PPI network analysis exhibited 45 points and 274 edges (Figure 3C), and the higher degree value resulted in larger and darker nodes. To identify the most critical targets within the network, we selected targets based on their degree values in the PPI network. The analysis revealed eleven core targets (Figure 3D), and three core targets (TLR4, NF-κB, and STAT3) were selected for further investigation due to their well-established roles in wound healing [7,8].

**Figure 4:**
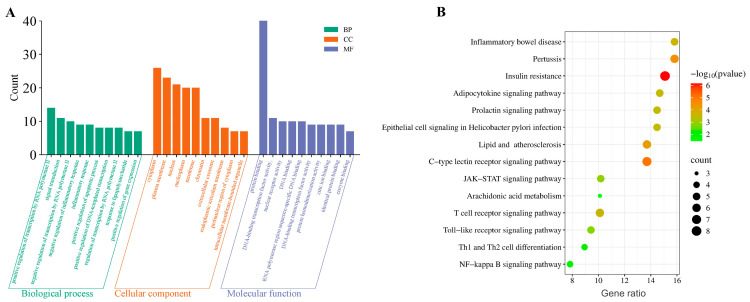
Analysis of GO and KEGG enrichments. (**A**) Top ten GO enrichment terms categorized by Biological Process (BP), Cellular Component (CC), and Molecular Function (MF). (**B**) Top fifteen KEGG pathways associated with wound healing.

The common targets were classified into 248 GO terms, of which 159, 29, and 60 GO terms belonged to BP, CC, and MF, respectively. The top ten GO terms were displayed according to count (Figure 4A), and they were enriched for BP including the positive regulation of RNA polymerase II transcription, negative regulation of inflammatory response, and signal transduction. The cytoplasm, nucleus, and plasma membrane were the main enrichments of CC while the MF of these targets mainly contained protein binding, DNA-binding transcription factor activity, and nuclear receptor activity. The top 15 pathways enriched by KEGG analysis are shown in Figure 4B, and the pathways with larger and darker bubbles might play crucial roles in specific biological processes. Insulin resistance was the most significantly enriched pathway, and this mainly affected wound healing through the cascade disturbance of inflammatory metabolism. Moreover, the NF-κB signaling pathway, JAK-STAT signaling pathway, and Toll-like receptor signaling pathway were also highly enriched, which was consistent with the predicted core targets of wound healing.

### 2.4. Molecular Docking Analysis

To evaluate the affinity of MDs to core proteins (TLR4, NF-κB, and STAT3), molecular docking was used. When the binding energy was less than zero, it meant that the interaction between the two molecules made the energy of the system lower. In other words, these two molecules could be bound together spontaneously without the need for additional energy from the outside. The strength of molecular binding ability and the stability of a conformation increased as the binding energy decreased. Computational docking exercises were performed to elucidate the characteristics of the ingredient–target binding mode. The results of binding energy (Table 2) indicated that the affinity of TLR4, NF-κB, and STAT3 with all ligands consistently remained below −6.0 kcal/mol. This suggests that the MDs exhibit significant binding activity with protein targets related to wound healing, and the credibility of the network pharmacology predictions was supported by these findings. To validate the docking protocol, a re-docking study with the co-crystallized ligands of each target was performed, resulting in RMSD values below 2 Å, confirming the reliability of the docking procedure. Each molecule demonstrated the strongest binding affinity for TLR4, followed by NF-κB and then STAT3. Comparative analysis with a well-characterized standard inhibitor showed that most MDs exhibited comparable or superior binding affinities, further supporting their potential as effective binders. Furthermore, the potent inhibitory activity of MDs is attributed to their engagement in strong hydrogen bonding, van der Waals forces, and hydrophobic interactions with key amino acid residues within the active site of the target protein (Table S2). All tested molecules interacted with TLR4 through a common binding site at residue CYS133. For NF-κB, the common binding residues included GLY55, GLY68, SER74, GLU76, SER75, LYS77, LYS79, SER81, and ASN250. In the case of STAT3, the shared interaction sites were located at GLU638, TYR657, and LYS658.

### 2.5. Reparative Effect In Vitro

The cell viability of MDs was measured to carry out subsequent experiments. As depicted in Figure S3, MDs (12.5, 25 µM) did not exhibit any statistically significant cytotoxic effects, and these were used for subsequent experiments. When the concentration of MDs exceeded 50 µM, the toxicity of MDs to HaCaT showed an initial enhancement followed by attenuation with an increase in the sugar base.

**Figure 5:**
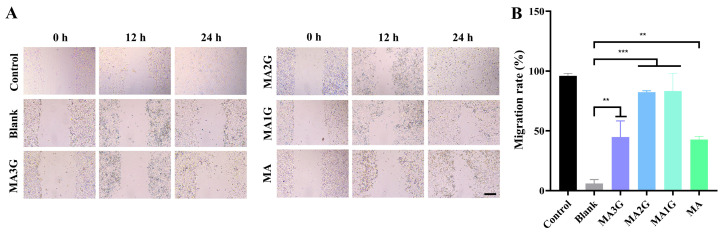
The effect of MDs on HaCaT migration. (**A**) Representative images of the scratch assay on HaCaT cells treated with MDs (25 µM) at selected time points (Scale bar: 200 µm ). (**B**) Quantification of migration rate in HaCaT cells at 24 h. Data are expressed as mean values ± SD (n = 3). ** *p* < 0.01, and *** *p* < 0.001 vs. blank group.

Keratinocytes constitute the primary cellular components of the epidermis and are often used in the study of skin damage repair [24]. The scratch assay in vitro was employed to estimate the healing effects of drugs on damaged skin. The results are shown in Figure 5A. The control group (complete medium) basically completed migration within 24 h while the blank group (serum-free medium) had negligible migration. Simultaneously, all tested MDs significantly enhanced the migration of human keratinocytes in vitro. Figure 5B shows that MA1G and MA2G displayed higher migratory capacity values, which were approximately 83.28% and 82.39%. In contrast, the MA3G (44.91%) and MA (42.76%) groups exhibited moderate promotional effects, which were significantly lower than those of MA1G and MA2G.

### 2.6. Antioxidant Effects

**Figure 6:**
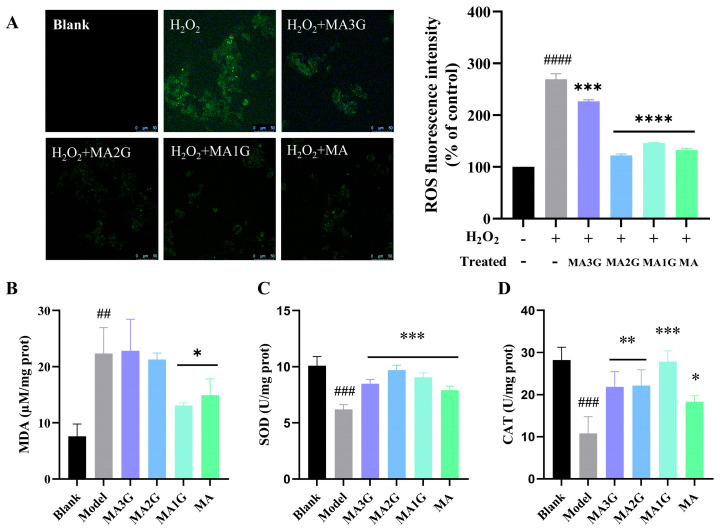
The effect of MDs on oxidative stress. (**A**) Fluorescence intensity of ROS in HaCaT cells treated with MDs (25 µM). (**B**–**D**) Contents of Malondialdehyde (MDA), Superoxide Dismutase (SOD), and Catalase (CAT) in HaCaT cells treated with MDs (25 µM), respectively. Data are expressed as mean values ± SD (n = 3). ## *p* < 0.01, ### *p* < 0.001, and #### *p* < 0.0001 vs. blank group; * *p* < 0.05, ** *p* < 0.01, *** *p* < 0.001, and **** *p* < 0.0001 vs. model group.

Excessive reactive oxygen species in the complex wound microenvironment can delay wound healing as they can further aggravate inflammation and damage angiogenesis [25]. The antioxidant capacity of MDs was verified through the utilization of the ROS scavenging assay. As shown in Figure 6A, H_2_O_2_ significantly increased ROS content in cells (2.70-fold, *p* < 0.001). Treatment with MDs greatly alleviated H_2_O_2_-induced ROS production, indicating that all MDs have a strong ability to scavenge ROS. In addition, the difference in fluorescence intensity between the model group and the MA3G group (2.26-fold) was minimal while MA2G (1.22-fold) and MA1G (1.46-fold) exhibited extremely strong ROS scavenging activity.

MDA, SOD, and CAT were further measured to explore the antioxidant effect of MDs. As shown in Figure 6B, only MA1G and MA can significantly reduce intracellular MDA levels, suggesting that MDA may not be the main mechanism of MDs against oxidative stress. SOD and CAT are important antioxidant substances that maintain the balance of oxidative stress in the body by scavenging free radicals. SOD and CAT levels decreased dramatically in cells exposed to H_2_O_2_ (Figure 6C,D), and antioxidant indicators levels increased significantly in cells treated with MDs. Moreover, it was found that MA2G and MA1G with moderate glycosyl exhibit better resistance to oxidative stress.

### 2.7. The Expressions of Core Targets

**Figure 7:**
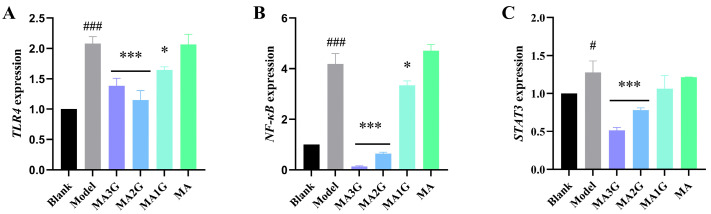
(**A**–**C**) The expression levels of Toll-like receptor 4 (TLR4), nuclear factor kappa B (NF-κB), and signal transducer and activator of transcription 3 (STAT3) in HaCaT cells were detected by RT-qPCR, respectively. Data are expressed as mean values ± SD (n = 3). # *p* < 0.05 and ### *p* < 0.001 vs. blank group; * *p* < 0.05 and *** *p* < 0.001 vs. model group.

To further verify the network pharmacology outcomes, the effects of MDs on the levels of TLR4, NF-κB, and STAT3 were tested. The H_2_O_2_-induced model groups exhibited significantly elevated expression levels of TLR4 (2.08-fold), NF-κB (4.19-fold), and STAT3 (1.36-fold) compared to the blank groups for each indicator. MA3G (1.38-fold) and MA2G (1.15-fold) exhibited a reduction in TLR4 mRNA expression, as illustrated in Figure 7A. Furthermore, MA3G (0.15-fold) and MA2G (0.64-fold) demonstrated an obvious decrease in NF-κB mRNA expression, followed by MA1G (Figure 7B). Remarkably, MA3G (0.51-fold) and MA2G (0.79-fold) also exhibited a substantial decrease in STAT3 mRNA expression induced by H_2_O_2_, (Figure 7C). These results suggest that the downregulation of TLR4, NF-κB, and STAT3 mRNA expression induced by MDs may contribute to the inhibition of ROS-mediated inflammation.

### 2.8. The Repair Effect of MDs In Vivo

**Figure 8:**
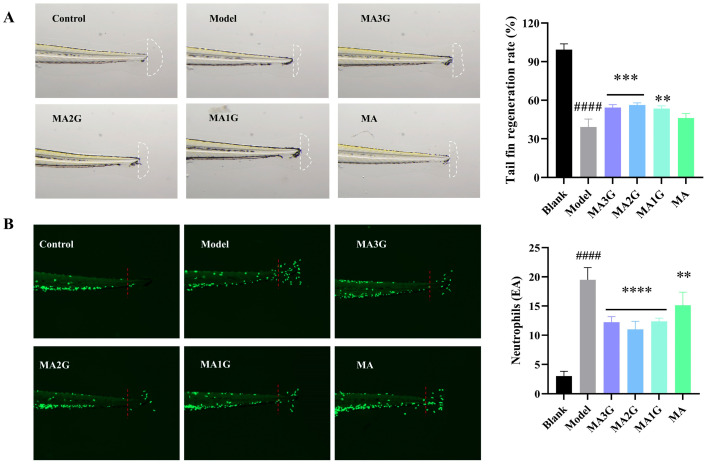
Effects of MDs on caudal fin regeneration and neutrophil migration in zebrafish. (**A**) Morphology and quantification of caudal fin regeneration rate in zebrafish following 48 h exposure to MDs (0.005%). (**B**) Image and quantitative analysis of neutrophil number (green fluorescence) in caudal fin zebrafish following 5 h exposure to MDs (0.005%). Data are expressed as mean values ± SD (n = 15). #### *p* < 0.0001 vs. blank group; ** *p* < 0.01, *** *p* < 0.001, and **** *p* < 0.0001vs model group.

The zebrafish is an ideal model for studying wound healing due to its powerful tissue regeneration ability. The tails fin regeneration of zebrafish involves multi-stage dynamic events such as inflammation regulation, cell dedifferentiation, proliferation, and morphological reconstruction, so the tail docking model of zebrafish was used to explore the wound repair effect of drugs in vivo [26]. In addition, researchers can also use genetically modified zebrafish to explore the regulatory effect of drugs on the inflammatory microenvironment [27]. The maximum tolerated concentrations (MTCs) of the MDs have been determined in zebrafish to ensure safety. MA1G, MA2G, and MA3G were found to be non-toxic at concentrations up to 0.05% (Table S3) whereas MA exhibited toxicity above 0.005%. Based on these results, a uniform concentration of 0.005% was used for all subsequent experiments. As depicted in Figure 8A, the model group exhibited significantly impaired caudal fin regeneration (39.13%), highlighting the detrimental role of oxidative inflammatory crosstalk in delaying wound healing. Pharmacological intervention with madecassoside derivatives (MA3G, MA2G, and MA1G) elevated regeneration rates to 53.66–56.34% while MA showed negligible efficacy. Consistently, neutrophil trafficking analysis (Figure 8B) revealed a robust neutrophil influx in the model group, consistent with unresolved inflammation. MDs treatment effectively suppressed neutrophil migration to the lesion area, further supporting the hypothesis that their tissue regeneration effects are mediated through the attenuation of inflammatory cascades.

## 3. Discussion

*Centella asiatica* (CA) has been used in ancient times to treat skin disease, and triterpenoids are thought to be key active compounds [28]. Previous studies showed that triterpenoids with diverse structures in CA have a different activity, and madecassoside has attracted extensive attention due to its unique structure and physiological activity [29]. Notably, madecassoside is metabolically converted in vivo to deglycosylated derivatives with enhanced bioavailability. However, there have been no systematic comparative studies on the madecassoside and its metabolites, mainly due to the unavailability of metabolites. The aim of this study was to prepare the deglycosylation products of madecassoside by a simple degradation inspired by metabolism and to evaluate its potential for the treatment of wound healing. Finally, the potential mechanism was elucidated through network pharmacology and experiments.

Firstly, the cytotoxicity of the four MDs was evaluated, and it was found that the deglycosylation of madecassoside attenuates its toxicity to HaCaT. In the scratch assay, all MDs could promote the migration of HaCaT. MA1G had the strongest migration-stimulating effect among all samples, followed by MA2G. This indicates that the proper glycosylation at C28 could contribute to the repair of damaged skin. Interestingly, although MA1G showed the strongest effect in cell migration, it was not consistently superior in other assays, suggesting that different aspects of wound healing (e.g., migration versus anti-inflammatory activity) may be modulated by distinct glycosylation patterns. This highlights the need to study stage-specific roles of MDs in wound healing. The difference in activity may have been due to a change in the planar structure and hydrophobicity of the compound after the introduction of the group [30].

ROS is considered to be one of the key factors influencing the pathological progression of wound healing. On the one hand, high levels of ROS directly impair skin cells, inhibiting their proliferative and migratory capacities; on the other hand, ROS activates immune cells, thereby inducing and aggravating the inflammatory response [31]. Hence, the H_2_O_2_-induced HaCaT cell skin lesion model was utilized. All MDs displayed ROS scavenging activity, indicating that MDs facilitate wound healing by oxidative stress reduction. However, MA3G has weaker ROS scavenging activity than other MDs, suggesting that the relatively short glycosyl chain was essential for enhancing the antioxidant activity of triterpenoid glycoside from CA, and other studies have also confirmed this conclusion [32,33]. This discrepancy between MA3G and other MDs may indicate that excessive glycosylation, while beneficial for solubility, compromises the compound’s ability to interact with ROS. Additionally, the type of monosaccharide is of great importance. In general, the antioxidant activity of O-glucoside is superior to that of O-arabinoside [21]. Our findings are in line with those of previous studies, which may imply that triterpenoid glycosides from CA also have a similar structure–activity relationship.

In the present study, network pharmacology was used to study the wound healing mechanism of MDs. The results of PPI indicated that NF-κB, TLR4, and STAT3 were identified as crucial targets for wound healing. Within the top KEGG pathways, the majority were associated with inflammation, immune response, and oxidative stress. To investigate the binding mode and affinity of MDs with STAT3, TLR4, and NF-κB, molecular docking was performed. The molecular docking results demonstrated that the MDs modulate TLR4 by interacting with CYS133, which aligned with the findings of Bai et al. [34]. Similarly, Bukhari et al. confirmed the anti-diabetic activity of butin through docking studies with NF-κB and also concluded that its inhibitory activity involved residues such as GLY55, GLY68, SER74, SER75, GLU76, LYS79, and SER81 [35]. Furthermore, our docking results with STAT3 corroborate the work of Wang et al., verifying that residues GLU638 and TYR657 are critical for the identification of STAT3 inhibitors [36]. The results indicated that the affinity of MDs with di- and tri-glycoside (MA2G and MA3G) to target proteins was stronger than that of MDs with non- and mono-glycoside (MA and MA1G). Jiang et al. found that the interactions of ICA (with two glycosyl groups) and BHG-I (with one glycosyl group) with protein targets were stronger than that of IT (without any glycosyl group), a finding that echoed the results of the present study [37]. However, it should be noted that docking results only suggest potential interactions under idealized in silico models and may not fully predict binding in the complex in vivo microenvironment. To further confirm the results of network pharmacology, we performed experiments in vitro. The results showed that excess ROS activated the STAT3/NF-κB pathway, which was consistent with the development of wound healing pathology, whereas TLR4, NF-κB, and STAT3 expression were significantly decreased after MD treatment. MA2G and MA3G exhibited extremely significant inhibitions of the expression of these core targets, which corresponded to the better molecular docking affinity of MA3G and MA2G. However, this only preliminarily indicates their qualitative correlation, and cannot clarify a clear quantitative relationship. Contrary to reports that O-glycosylation diminishes anti-inflammatory activity in flavonoids [38], our data demonstrated enhanced efficacy in highly glycosylated triterpenoids (MA2G/MA3G). This discrepancy may have arisen from fundamental structural differences, such as the effect of glycosylation on steric hindrance or hydrogen bonding, which could have modulated the bioactivity of these compounds. However, the specific structural mechanisms underlying remain to be further explored in future studies.

Furthermore, penetration through the skin barrier to reach the target site is essential for the therapeutic efficacy of drugs, where drugs with molecular weights < 600 Da, log P values of 1.0–3.0, and melting points < 200 °C exhibit enhanced permeation [39]. The results of this study demonstrated that moderately glycosylated MA1G (with one glycosyl group, Log P 2.64, *M*_w_ 667 Da) and MA2G (with two glycosyl groups, Log P 1.07, *M*_w_ 829 Da) significantly improved transdermal kinetics by lowering the melting point and optimizing Log P, which enhanced lipophilicity and skin permeability. In contrast, unglycosylated MA (Log P 3.97, *M*_w_ 505 Da) exhibited osmotic lag due to its high melting point and high Log P, which hindered its transdermal penetration. Highly glycosylated MA3G (with three glycosyl groups, Log P −0.56, *M*_w_ 975 Da) showed reduced early osmotic efficiency due to its low Log P and high molecular weight, which limited its ability to penetrate the skin effectively. Similarly, studies have demonstrated that glycosylation modification can significantly enhance the skin permeation properties of epigallocatechin gallate and naringin, which was highly consistent with the results of the present study [40,41]. These findings emphasize the critical role of glycosylation in modifying drug structure for optimized transdermal formulation design. However, it is important to note that Start-M, being an artificial membrane, may not fully replicate the characteristics of human skin, which could limit the accuracy of permeability predictions in vivo.

Given its balanced bioactivity, target affinity, and transdermal properties, MA2G emerges as a promising candidate for targeted therapeutic applications in wound healing. Despite these promising findings, several limitations should be acknowledged. The use of zebrafish as a model, rather than mammalian validation, limits the ability to fully assess the compounds’ efficacy and safety in a more complex biological system. Additionally, future studies should focus on acquiring comprehensive pharmacokinetic profiles to support the clinical development of these compounds.

## 4. Materials and Methods

### 4.1. Materials and Chemicals

Madecassoside (MA3G) and madecassic acid (MA) were obtained by NatureStandard (Purity ≥ 95%, Shanghai, China). Madecassoside biotransformed metabolites (MA2G and MA1G) were gained by enzymatic hydrolysis, and the detailed procedures are provided in the Supplementary Materials. SOD, MDA, CAT, and GSH assay kits were purchased from Beyotime Biotechnology (Shanghai, China).

### 4.2. Network Pharmacology

The targets of MDs were acquired from TCMSP, SwissTargetPrediction, and PharmMapper databases, and the repeated target was deleted [30]. The wound-healing-related targets were obtained by the GeneCards database DrugBank, OMIM, and TTD databases [42]. Venny2.1.0 was used to analyze targets shared between MDs and wound healing. Then, MDs were arranged according to the numbers of disease-related target genes. The data of intersecting targets was inputted into Cytoscape 3.9. software to form a “MDs-target-wound healing’’ network [43]. The STRING database was employed to analyze protein interactions and Cytoscape was utilized to visualize the PPI network. Gene Ontology (GO) and Kyoto Encyclopedia of Genes and Genomes (KEGG) analysis were applied for the enrichment of functional classification of protein targets [44].

### 4.3. Molecular Docking

The protein structures of STAT3 (PDB ID: 6NJS) [45], TLR4 (PDB ID: 3FXI) [46] and NF-κB (PDB ID: 1SVC) [35] were obtained from the RCSB Protein Data Bank (RCSB PDB). Vitexin was used as the positive control for TLR4 and NF-κB while PDB ID: KQV was used as positive control for STAT3. Ligand structures were constructed using ChemOffice and subsequently energy-minimized. Molecular docking was carried out using AutoDock Vina (version 1.1.2) with a grid box size of 40 × 40 × 40 Å centered on the active site of each target protein. The resulting docking poses were visualized using Discovery Studio 2019 to generate 2D and 3D interaction diagrams. All structures were validated prior to analysis, with root mean square deviation (RMSD) values of less than 2 Å.

### 4.4. Preparation and Identification of MA1G and MA2G

See Supplementary Materials.

### 4.5. Skin Permeation Test Studies

See Supplementary Materials.

### 4.6. Cell Viability Assay

The immortalized human keratinocyte cell line HaCaT (BNCC, Xinyang, China) was cultured in DMEM with 10% FBS and 1% penicillin–streptomycin at 37 °C in 5% CO_2_, and the cells within 25 passages were used for subsequent experiments. The cells were cultured in 96-well plates for 24 h. The MDs were added to the cells to screen out the suitable concentrations, and PBS served as the control. The cell viability was tested by MTT assay [47]. The measurement was performed at 490 nm. Each concentration sample group and control group were equipped with six parallel wells.

### 4.7. Scratch Wound Assay

The cells (3.5 × 10^5^ cells/mL) were seeded in culture inserts (ibidi, Gräfelfing, Germany) for 12 h [48]. The inserts were removed and PBS was used to wash the cells. The DMEM (blank), DMEM containing MDs, and DMEM containing FBS (positive control) were added to the cells. The scratched area was photographed at 0 h and 24 h, and the scratch area was calculated using ImageJ 1.52i software. The migration rate was assessed by calculating the percentage of the initial wound area covered after 24 h.

### 4.8. Reactive Oxygen Species Assay

HaCaT cells were seeded in dishes, and the MDs (25 µM) were added to cells for 1 h pretreatment, and then we introduced hydrogen peroxide (H_2_O_2_, 500 µM) into the MDs and model group. After incubating for 24 h, the cells were washed twice with PBS. DCFH-DA solution was then added and incubated for 30 min, followed by two additional washes with PBS. Fluorescence images were captured using confocal laser scanning microscopy (CLSM) with excitation at 488 nm.

### 4.9. Detection of Biochemical Indicators of Oxidative Stress

HaCaT cells were seeded in six-well plates, and pretreated cells with DMEM containing MDs (25 μM) for 1 h, and then added with H_2_O_2_ (500 μM). The cells were collected and lysed, and the supernatant prepared by centrifugation was used for subsequent detection after 24 h culturing. SOD, CAT, and MDA were measured following kit instructions.

### 4.10. RT-qPCR Analysis

The RNA was extracted using trizol, and RT-qPCR measurements were carried out utilizing the SYBR Green Master Mix. Relative mRNA levels were quantified employing the 2^−ΔΔCt^ method and normalized to β-actin [49]. The primer sequences are shown in Table S1.

### 4.11. Healing Effect of MDs on Zebrafish

Wild-type AB strain zebrafish at 3 days post fertilization were selected and randomly distributed into 6-well plates at a density of 15 larvae per well. The caudal fin in the control group remained intact whereas a sterilized scalpel was used to amputate the caudal fin in the model and sample groups. Three experimental groups were established: (1) control group (untreated), (2) model group (fin amputation + culture medium), and (3) sample group (fin amputation + MDs). Then the zebrafish were treated with 0.005% MDs (sample group) or with culture medium (control and model groups). After 48 h, caudal fin morphology was recorded under a microscope, and the regenerated fin area in each group was quantified using ImageJ software. The regeneration rate of the caudal fin was assessed by calculating the percentage of the regenerated fin area relative to the control group after 48 h. All animal procedures were conducted in accordance with the guidelines of the Institutional Animal Care and Use Committee (License Number: IACUC-2023-7475-01).

### 4.12. The Anti-Inflammatory Effect of MDs on Zebrafish

The zebrafish were grouped and cultured according to Section 4.11, and the samples were incubated with zebrafish in the dark for 5 h, and then the number of neutrophils in the caudal fin region was analyzed by taking pictures under a fluorescence microscope. Neutrophil counts in the zebrafish tail were performed using ImageJ software.

### 4.13. Statistical Analysis

All data are expressed as the mean ± standard deviation (SD) values of three biological replicates. Statistical analysis was performed using one-way analysis of variance (ANOVA) with GraphPad Prism 9.5., and *p* < 0.05 was considered statistically significant.

## 5. Conclusions

In this study, metabolites of madecassoside (MA2G and MA1G) were prepared by a simple enzymatic digestion strategy, and the biological activities of madecassoside (parent compound) and its metabolites were comparatively investigated for the first time. Our findings revealed that the glycosyl number of MDs affected their antioxidant and anti-inflammatory activities, with MA2G showing comparatively stronger effects. Network pharmacology analysis further indicated that NF-κB and STAT3 signaling were key pathways through which MDs regulated wound healing. These findings provide preliminary mechanistic insights into the wound healing potential of MDs and highlight MA2G as a promising candidate for further evaluation. This work also facilitates the development of structure-specific triterpenoid derivatives for targeted dermatological applications. Future studies should focus on validation in mouse wound models and the development of advanced delivery formulations to accelerate clinical translation.

## Figures and Tables

**Table 1:** Identified compounds of MA2G and MA1G by LC-MS.

Samples	Formula	R_t_ (min)	Found Mass (*m*/*z*)	MS/MS (*m*/*z*)
MA2G	C_42_H_68_O_16_	7.53	829 [M + H]^+^	649, 487
MA1G	C_36_H_58_O_11_	8.28	667 [M + H]^+^	505, 487, 405

**Table 2:** Docking binding energies and RMSD of MDs and positive control.

Targets	Compounds	Binding Energy (kcal/mol)	RMSD (Å)
TLR4	MA	−8.6	0.0202
	MA1G	−8.7	0.2501
	MA2G	−8.2	0.0386
	MA3G	−9.6	0.1914
	Positive control	−7.4	0.0043
NF-κB	MA	−7.1	0.0484
	MA1G	−7.2	0.0301
	MA2G	−7.6	0.2153
	MA3G	−8.9	0.0289
	Positive control	−6.6	0.0153
STAT3	MA	−6.5	0.1072
	MA1G	−6.6	0.2085
	MA2G	−6.7	0.1806
	MA3G	−7.3	0.2047
	Positive control	−9.0	0.1918

## Data Availability

Data is contained within the article.

## Supplementary Materials

The following supporting information can be downloaded at: https://www.mdpi.com/article/10.3390/ph18091292/s1, Figure S1. The mass spectra of MA2G and MA1G obtained by LC-MS using BEH C18 column with 0.1% formic acid and 0.1% ammonium acetate in water and acetonitrile as mobile phase; Figure S2. The HPLC chromatogram of MDs using Acclaim™ 120 C18 column with water and acetonitrile as mobile phase; Figure S3. Cell viability of HaCaT cells treated with MDs (12.5, 25, 50, and 100 µM) for 24 h performed by MTT assay; Table S1. Primer sequence of RT-qPCR reaction system; Table S2. Binding sites of MDs for three targets; Table S3. Results of the maximum tolerated concentration of MDs in zebrafish (n = 30).
