# Supplementary material for: Unraveling the Mechanisms of Madecassoside Derivatives in Wound Healing: Network Pharmacology and Experimental Validation
Source: Pharmaceuticals (Basel). 2025 Aug 28;18(9):1292. doi: 10.3390/ph18091292 (PMC12472257; doi:10.3390/ph18091292)
Supplement: Supplementary file 1 [file pharmaceuticals-18-01292-s001.zip › pharmaceuticals-3830951-supplementary.pdf]

Supplementary Information

Unraveling the Mechanisms of Madecassoside Derivatives in Wound Healing: Network Pharmacology and Experimental Validation

Jing Liu, Yuanyuan Li, Cheng Yang * and Bingtian Zhao *

Key Laboratory of Synthetic and Biological Colloids, Ministry of Education, School of Chemical and Material Engineering, Jiangnan University, Wuxi 214122, China; lj15735649029@163.com (J.L.); liyuanyuan0918@163.com (Y.L.)

* Correspondence: cyang@jiangnan.edu.cn (C.Y.); btzhao@jiangnan.edu.cn (B.Z.)

1. The Preparation of MA1G and MA2G

15 g of MA3G was dissolved in 900 mL of 4 mM Citric acid - Sodium citrate Buffer (pH = 4.5), and then 2.5 g of α-L-rhamnosidase (Yukong Biotechnology Co., Ltd., Zhengzhou, 40 kU) was added for hydrolysis 24 h at 60 ℃. The enzyme activity was inactivated by high pressure and filtration was carried out. Subsequently, it was evaporated to dryness, and 12.5 g of MA2G was obtained. MA1G was obtained (10.5 g) by hydrolysis with 0.5 g cellobiase (Yukong Biotechnology Co., Ltd., Zhengzhou, 1 kU) added on the above basis.

2. Identification of MA1G and MA2G

The sample was identified by liquid chromatography mass spectrometry (Waters MALDI SYNAPT MS) equipped with BEH C18 column (2.1 × 100 mm, 1.7 μm). The mobile phase consisted of 0.1% formic acid and 0.1 % ammonium acetate in water and acetonitrile (60:40, v/v), with 0.3 mL/min flow rate. The ion source is an electrospray ion source, with positive ion detection. Capillary voltage: 3.5 kV; Ion source temperature: 100 °C; Desolvation temperature: 400 °C; Cone voltage: 50 V; Collision energy: 6 V; Detection voltage: 1800 V; The full scan mass range is set to 50 - 2000 m/z.

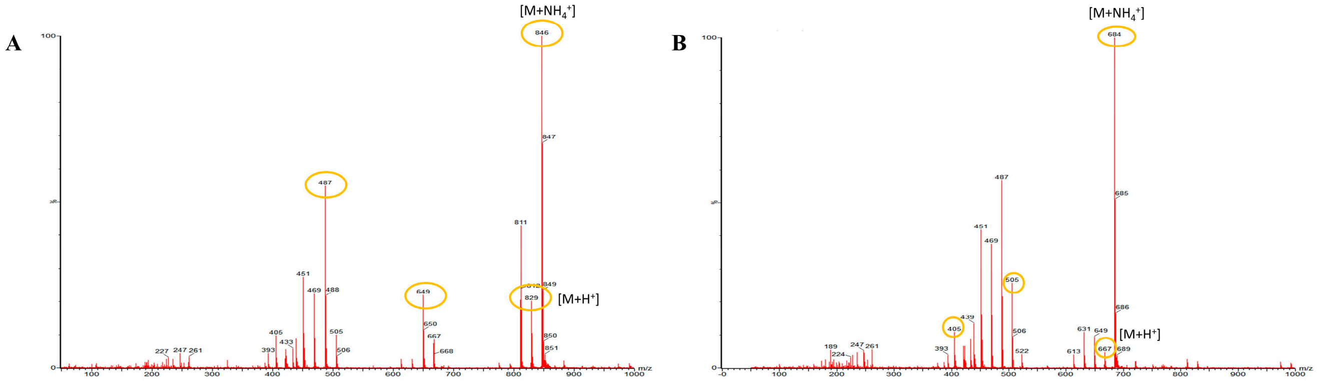

Figure S1. The mass spectra of MA2G (A) and MA1G (B) obtained by LC-MS using BEH C18 column with 0.1 formic acid and 0.1 % ammonium acetate in water (A) and acetonitrile (B) as mobile phase.

3. Purity detection by HPLC

1 mg of samples were respectively dissolved in 1 mL of chromatographic methanol, and filter it through a 0.45 μm organic syringe filter to obtain the solution. The chromatographic column is an Acclaim™ 120 C18 (4.6 × 250 mm, 5.0 μm); the mobile phase is water (A) and acetonitrile (B); the binary gradient conditions: A and B from 75:25 to 35:65 for 60 min, from 35:65 to 75:25 for 10 min at a flow rate 1 mL/min; the detection wave length is 205 nm. The chromatography of each component shown in Figure S2. Among them, MA3G, MA, SA3G, and SA are positive standard products for market acceptance.

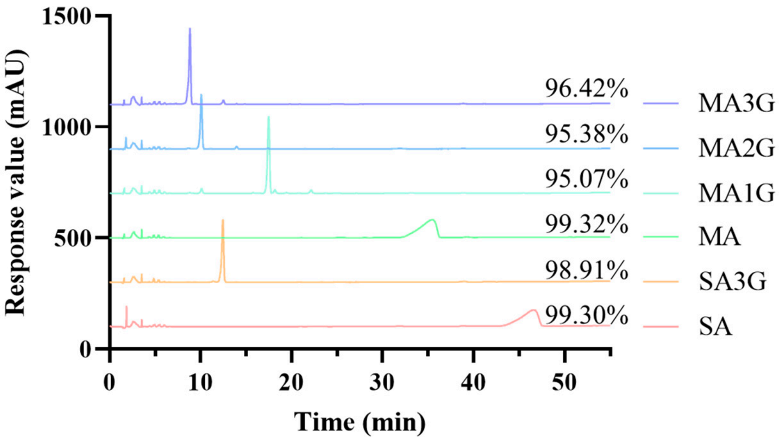

Figure S2. The HPLC chromatogram of MDs using Acclaim™ 120 C18 column with water (A) and acetonitrile (B) as mobile phase. MA3G: Madecassoside, MA2G: Centellasaponin B, MA1G: O- glucopyranosyl-2,3,6,23-tetrahydroxyurs-12-en-28-oate, MA: Madecassic acid, SA3G: Asiaticoside, and SA: Asiatic acid.

4. Skin permeation test studies *in vitro*

The penetration test was performed using a static vertical glass Franz diffusion cell with a diffusion area of 3.14 cm2 and a volume of 1.0 and 8.0 mL for the donor and acceptor, respectively. The Strat-M® membrane was sandwiched between Franz's donor and recipient compartments, and a 1250 μg/mL MDs solution was added to the donor chamber and sealed with parafilm. A magnetic rotor was added to the receiving cell, and the whole system was placed in a transdermal diffuser at a constant speed of agitation and constant temperature, at 37 °C, and at a rotational speed of 500 r/min. 0.2 mL of the receiving solution was collected at a specific time, the collected solution was centrifuged at 12000 r/min for 10 min, the supernatant was taken and the concentration of the drug was analysed by HPLC, and the cumulative transdermal volume (Qn, μg) of MDs at each time point was calculated using equation (1):

$$Q_n = VC_n + \sum_{i=1}^{i=n-1} V_s C_i$$

With V the volume of receiving chamber, V_s the sample volume, C_n the concentration of the drug in the receiving solution measured at the nth sampling point, C_i Drug concentration at sampling point i.

5. Determination of Maximum Tolerated Concentration (MTC) in Zebrafish

Wild-type AB zebrafish larvae at 3 days post-fertilization (dpf) were used to determine the maximum tolerated concentration (MTC) of the MDs. Zebrafish larvae were randomly distributed into 6-well plates at a density of 30 larvae per well, and they were divided into a blank control group and sample groups with different concentrations. After 48 h of exposure, larval survival was recorded, and the MTC for each compound was defined as the highest concentration that did not cause significant mortality. Detailed concentration ranges and survival data are provided in Table S3.

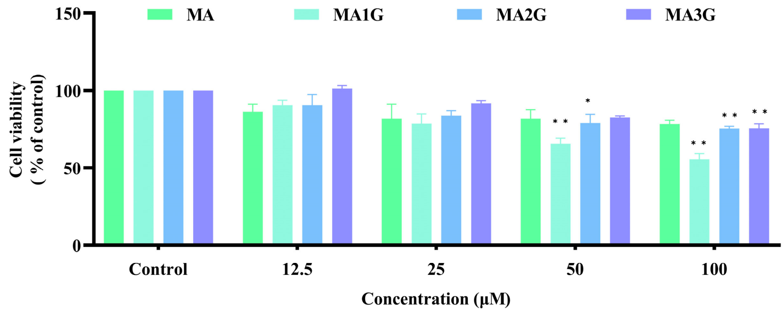

Figure S3. Cell viability of HaCaT cells treated with MDs (12.5, 25, 50, 100 μM) for 24 h performed by MTT assay. Data expressed as mean values ± SD (n=3). *p < 0.05, and **p < 0.01 vs Control group.

Table S1. Primer sequence of RT-qPCR reaction system.

Gene	Primer	Sequence (5'→3')	Length (bp)
Homo β-actin	Forward	CCCTGGAGAAGAGCTACGAG	180
	Reverse	CGTACAGGTCTTTGCGGATG	
Homo STAT3	Forward	TTTTGTCAGCGATGGAGTA	170
	Reverse	TTGTTGACGGGTCTGAAGT	
Homo TLR4	Forward	ACCTGTCCCTGAACCCTAT	135
	Reverse	CTAAACCAGCCAGACCTTG	
Homo NF-κB	Forward	GATTTCGTTTCCGTTATG	120
	Reverse	TTGCTGGTCCCACATAG	

Table S2. Binding sites of MDs and positive control for three targets.

Compounds		Targets	H-Bonds interacting residues	Van der Waals interacting residues
MA		TLR4		ILE46, LEU54, LEU61, PHE119, PHE126, CYS133
		NF-κB	LYS52, SER74, GLU76	**GLY55**, PHE56, **GLY68**, GLY69, SER75, LYS77, ASN78, LYS79, LYS80, SER81, ASN250
		STAT3	PRO639, GLN644, **TYR657**	GLU638, THR641, **LYS658**, ILE659, MET660
MA1G		TLR4		LEU54, LEU61, LEU78, ILE80, LEU87, ARG90, CYS133, PHE126
		NF-κB	SER75	**GLY55**, ARG59, HIS67, **GLY68**, SER74, GLU76, LYS77, LYS79, SER81, ASN250
		STAT3	GLU638, GLN644, **TYR657**	VAL637, THR641, **LYS658**, ILE659, LEU666
MA2G		TLR4	CYS133	ARG264; VAL24, ILE32, ILE46, VAL48, ILE52, ILE63, LEU78, ILE80, TYR102, ILE117, SER118, SER120, TYR131, PHE151, ILE153
		NF-κB	LYS52, ARG59, SER74, GLU76, LYS79, SER243, ASN250	**GLY55**, ARG57, **GLY68**, GLY69, SER75, LYS77, ASN78, LYS80, SER81, LYS252
		STAT3	GLU638, ASN647, **TYR657**, TYR640	TRP623, MET648, ILE653, **LYS658**
MA3G		TLR4	ARG264	SER317, ASN339, LYS363; VAL48, LEU54, ASP100, ASP101, TYR102, PHE104, PHE119, PHE121, CYS133， PHE151
		NF-κB	LYS52, LYS79, ASN250, LYS252	**GLY55**, **GLY68**, GLY69, SER74, SER75, GLU76, LYS77, ASN78, LYS80, SER81, LYS244, SER249, ASP274
		STAT3	GLU638, TYR640, **TYR657**	GLU625, PRO639, THR641, GLN644, MET648, ILE653, GLY656, **LYS658**, ILE659, LEU666
Positive control	Vitexin	TLR4	ASP294	TYR296, SER317, THR319, ARG264, ASN339; C: ASP101, TYR102, SER118, PHE119, SER120, ARG264
		NF-κB	**GLY55**, ARG59, HIS67, ASN139,	PHE56, ARG57, **GLY68**, **GLY69**, LYS80, TYP82, SER243
	KQV	STAT3	SER611, GLU612, SER613, SER636, **TYR657**	SER614, THR620, TRP632, GLN635, **GLN644**, **LYS658**, **ILE659**, MET660

Note: red for those residues commonly interacting with the MDs, and blue for those forming interactions with both the MDs and positive control.

Table S3. Results of the maximum tolerated concentration of MDs in zebrafish (n=30).

Group	Concentration (%)	Deaths / larvae	Mortality rate (%)
Blank	0	0	0
	0.005	0	0
MA3G	0.01	0	0
	0.05	0	0
	0.005	0	0
MA2G	0.01	0	0
	0.05	0	0
	0.005	0	0
MA1G	0.01	0	0
	0.05	0	0
	0.005	0	0
MA	0.01	30	100
	0.05	30	100
